# Social determinants of violence against women and associated health consequences in Latin America and the Caribbean: a scoping review

**DOI:** 10.1590/0102-311XEN048426

**Published:** 2026-10-30

**Authors:** Ana María Arias-Uriona, Paola Bedoya, Kate McGurn Centellas, Andrés Villalobos, Jazmín Mazó, Marcela Losantos

**Affiliations:** 1 Instituto de Investigaciones en Ciencias del Comportamiento, Universidad Católica Boliviana “San Pablo”, La Paz, Bolivia.; 2 Facultad de Derecho y Ciencia Política, Fundación Universitaria Los Libertadores, Bogotá, Colombia.; 3 Department of Sociology and Anthropology, University of Mississippi, University, U.S.A.

**Keywords:** Social Determinants of Health, Gender-Based Violence, Violence Against Women, Mental Health, Determinantes Sociales de la Salud, Violencia de Género, Violencia Contra la Mujer, Salud Mental, Determinantes Sociais da Saúde, Violência de Gênero, Violência Contra a Mulher, Saúde Mental

## Abstract

This scoping review examines how social determinants of health have been conceptualized and analyzed in relation to violence against women and its physical and mental health consequences in Latin America and the Caribbean. A structured bilingual search was conducted in PubMed/MEDLINE, Scopus, and SciELO for studies published between 2012 and 2025. In total, 82 empirical studies met eligibility criteria. Data were synthesized using a framework that distinguishes structural determinants, such as income inequality, racialized hierarchies, territorial segregation, gender norms, and labor precarity, from intermediary determinants, including age, relationship status, partner behaviors, material conditions, and social capital. The evidence demonstrates that violence against women in the region is socially patterned and structurally embedded. Economic inequality, poverty, and educational differentials within couples were consistently associated with exposure to violence, while intersecting systems of skin color, ethnicity, and territorial marginalization amplified risk. Intermediary mechanisms − including alcohol use, relational instability, childhood exposure to violence, and neighborhood deprivation − provided pathways linking these structural conditions to violence exposure. Health consequences were substantial and cumulative. Violence was strongly associated with depression, anxiety, post-traumatic stress symptoms, suicidal ideation, adverse maternal outcomes, reduced prenatal care utilization, and markers of cardiovascular risk. The findings indicate that violence against women in Latin America and the Caribbean constitutes a continuum of social and health inequality shaped by interacting structural and intermediary determinants. By organizing the regional evidence through a social determinants framework and incorporating an intersectional lens, this review advances a structurally grounded synthesis that clarifies how violence becomes socially produced and biologically embodied across the life course.

## Abstract (es)

Esta revisión exploratoria analiza cómo se han conceptualizado y analizado los determinantes sociales de la salud en relación con la violencia contra las mujeres y sus consecuencias para la salud física y mental en América Latina y el Caribe. Se realizó una búsqueda bilingüe estructurada en PubMed/MEDLINE, Scopus y SciELO para identificar estudios publicados entre 2012 y 2025. Ochenta y dos estudios empíricos cumplieron con los criterios de elegibilidad. Los datos se sintetizaron utilizando un marco de determinantes sociales de la salud que distingue entre los determinantes estructurales, como la desigualdad de ingresos, las jerarquías basadas en la raza, la segregación territorial, las normas de género y la precariedad laboral; y los determinantes intermedios, entre los que se incluyen la edad, el estado civil, los comportamientos de la pareja, las condiciones materiales y el capital social. Las pruebas demuestran que la violencia contra las mujeres en la región sigue patrones sociales y está arraigada estructuralmente. La desigualdad económica, la pobreza y las diferencias educativas entre las parejas, la exclusión por motivos raciales y la marginación territorial se asociaron de manera consistente con un mayor riesgo. Los mecanismos intermedios -entre ellos, el consumo de alcohol, la inestabilidad en las relaciones, la exposición a la violencia durante la infancia y las condiciones de privación en el vecindario- actuaron como mediadores de las condiciones estructurales. Las consecuencias para la salud fueron sustanciales y acumulativas. La violencia contra las mujeres se asoció fuertemente con depresión, ansiedad, síntomas de estrés postraumático, ideas suicidas, resultados maternos adversos, menor uso de la atención prenatal y marcadores de riesgo cardiovascular.

## Abstract (pt)

Esta revisão de escopo examina como os determinantes sociais da saúde foram conceituados e analisados em relação à violência contra as mulheres e suas consequências à saúde física e mental na América Latina e no Caribe. Uma busca bilíngue estruturada foi realizada nas plataformas PubMed/MEDLINE, Scopus e SciELO por estudos publicados entre 2012 e 2025. No todo, 82 estudos empíricos atenderam aos critérios de elegibilidade. Os dados foram sintetizados por um arcabouço de determinantes sociais da saúde que distingue determinantes estruturais (como desigualdade de renda, hierarquias racializadas, segregação territorial, normas de gênero e precariedade no trabalho) dos intermediários (incluindo idade, *status* de relacionamento, comportamento do consorte, condições materiais e capital social). As evidências demonstram que a violência contra as mulheres é socialmente padronizada e estruturalmente enraizada na região. Desigualdade econômica, pobreza e diferenciais educacionais dentro dos casais, exclusão racializada e marginalização territorial foram consistentemente associados a um risco maior. Mecanismos intermediários − incluindo uso de álcool, instabilidade relacional, exposição infantil à violência e privação de vizinhanças − mediaram essas condições estruturais. As consequências para a saúde foram substanciais e cumulativas. A violência contra as mulheres foi fortemente associada à depressão, ansiedade, sintomas de estresse pós-traumático, ideação suicida, desfechos maternos adversos, redução do uso do cuidado pré-natal e marcadores de risco cardiovascular.

## Introduction

Violence against women constitutes a sustained violation of human rights and a major public health concern. Globally, approximately one in three women experiences physical and/or sexual violence during their lifetime ^1^ ^,^ ^2^ ^,^ ^3^. In Latin America and the Caribbean, prevalence remains persistently high (25%), positioning the region among those bearing a disproportionate burden of gender-based violence ^1^ ^,^ ^2^ ^,^ ^3^. Intimate partner violence, sexual violence, and other forms of abuse continue to compromise women’s physical and mental health, autonomy, and social participation, with particularly acute effects among Indigenous and Afro-descendant women whose experiences remain underrepresented in epidemiological analyses and policy responses ^1^ ^,^ ^2^ ^,^ ^4^.

According to the United Nations *Declaration on the Elimination of Violence against Women* ^5^, violence against women encompasses any act of gender-based violence resulting in physical, sexual, or psychological harm that occurs within the family or community or is perpetrated or condoned by the state. In Latin America and the Caribbean, the persistence of such violence reflects entrenched gender regimes, pronounced socioeconomic stratification, racialized hierarchies, and institutional fragility, all of which reproduce structural disadvantage across generations ^1^ ^,^ ^2^ ^,^ ^3^ ^,^ ^4^.

The COVID-19 pandemic intensified pre-existing vulnerabilities, generating what has been described as a “shadow pandemic” of violence against women. Confinement measures and mobility restrictions heightened women’s exposure to abusive partners while simultaneously constraining access to support, justice systems, and health services ^6^ ^,^ ^7^. These dynamics exposed the structural nature of violence and underscored the necessity of embedding prevention and response strategies within broader public health and social protection systems.

The Social Determinants of Health (SDH) framework offers a robust analytical lens to interrogate these processes. By distinguishing structural determinants − such as governance arrangements, macroeconomic policies, labor market structures, gender norms, and social stratification − from intermediary determinants including material living conditions, psychosocial stressors, behavioral patterns, and access to services, the SDH model enables examination of how violence is socially patterned and biologically embodied ^8^ ^,^ ^9^ ^,^ ^10^ ^,^ ^11^. Applied to violence against women in Latin America and the Caribbean, this framework shifts analysis beyond individualized risk factors toward the social arrangements that structure exposure, constrain protection, and mediate institutional response.

Despite this conceptual relevance, the systematic application of SDH frameworks to violence against women research in Latin America and the Caribbean remains fragmented. Much of the existing literature emphasizes proximal correlates at the individual or household level, often without explicit theorization of structural pathways or examination of how determinants operate across intersecting axes of inequality ^4^ ^,^ ^12^ ^,^ ^13^ ^,^ ^14^ ^,^ ^15^.

This scoping review addresses these gaps by systematically examining how SDH have been conceptualized, operationalized, and empirically analyzed in relation to violence against women and its health consequences in Latin America and the Caribbean. Grounded explicitly in the SDH framework, the review maps structural and intermediary determinants, analyzes how these are linked to health outcomes, and identifies conceptual and methodological blind spots. An intersectional lens ^16^ was applied to assess whether and how studies addressed overlapping axes of inequality − including gender, skin color, ethnicity, socioeconomic status, age, and territorial marginalization − in shaping exposure to violence and health outcomes. By situating violence within the region’s context, this review contributes to a more structurally informed and policy-relevant evidence base.

### Review questions

1) How have structural and intermediary social determinants of health been conceptualized, operationalized, and empirically analyzed in relation to violence against women in Latin America and the Caribbean?

2) How do these determinants, individually and intersectionally, shape the physical and mental health consequences of violence against women in the region?

## Methods

### Study design

Given the heterogeneity of study designs, theoretical approaches, measurement strategies, and outcome definitions across the field, a scoping methodology was considered appropriate to map existing evidence, identify conceptual and methodological gaps, and clarify how determinants are positioned within empirical analyses.

The review followed the methodological guidance proposed by Armstrong et al. ^17^ and Peters et al. ^18^. Reporting adhered to the *Preferred Reporting Items for Systematic reviews and Meta-Analyses Extension for Scoping Reviews* (PRISMA-ScR) 2020 checklist, and the protocol was prospectively registered in the Open Science Framework (OSF; https://osf.io/35gpj/).

### Search strategy and study selection

A structured, bilingual (English and Spanish) search strategy was developed and organized into conceptual blocks corresponding to each review question. Searches were conducted in PubMed/MEDLINE, Scopus, and SciELO to ensure coverage of international and regionally indexed journals. The search period spanned from January 2012 to October 2025.

The full database-specific search strategies, including complete Boolean strings and country-level terms, are provided in the Supplementary Material (https://cadernos.ensp.fiocruz.br/ojs/index.php/csp/article/view/12914/21140) to ensure reproducibility and transparency.

The initial search yielded 3,295 records − of which 2,390 addressed Review Question 1, and 905 addressed Review Question 2 (Figure 1). All records were imported into Rayyan for duplicate identification and blinded screening.

**Figure 1:**
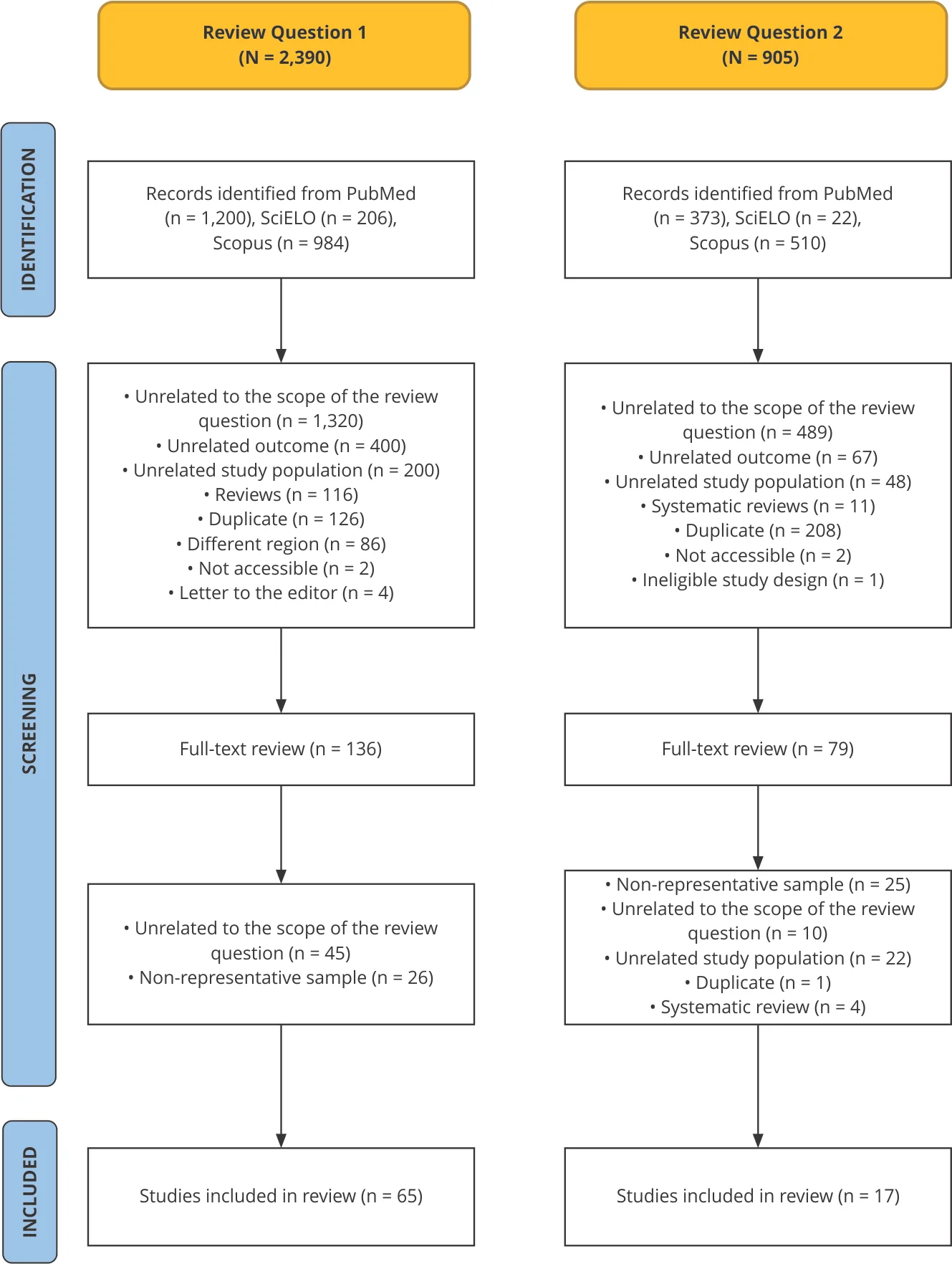
PRISMA flow diagram for the scoping review.

Study selection comprised title and abstract screening, followed by full-text review. Screening was conducted independently by three reviewers.

### Eligibility criteria

Studies were eligible for inclusion if they met the following criteria: (1) Reported original empirical data (quantitative, qualitative, or mixed-methods) on violence against women in countries of Latin America and the Caribbean; (2) Analyzed structural and/or intermediary social determinants of health in relation to violence against women, either as primary exposures, mediators, contextual variables, or stratifying factors; (3) Analyzed physical or mental health outcomes associated with violence; and (4) Were published in peer-reviewed journals in English or Spanish between January 2012 and October 2025.

Quantitative studies were included when they clearly described study design, sampling strategy, and measurement of violence and/or health outcomes. Qualitative studies were eligible when they used explicit methodological approaches and provided contextual insight into structural or intermediary determinants shaping violence and its health consequences.

Studies focusing exclusively on specific populations such as LGBTQ+ individuals, sex workers, or displaced populations were excluded due to differences in structural configurations, legal frameworks, and forms of violence affecting these groups, which warrant dedicated, context-specific syntheses rather than secondary inclusion within a broader review.

### Data extraction and analytical strategy

Extracted information included authorship, year of publication, country, study design, population characteristics, form(s) of violence examined, social determinants analyzed, health outcomes assessed, analytical approach and main findings (Supplementary Material, https://cadernos.ensp.fiocruz.br/ojs/index.php/csp/article/view/12914/21140).

Data synthesis combined descriptive mapping with a theoretically informed thematic analysis structured around the social determinants of health framework. An intersectional lens was used to examine how studies addressed overlapping axes of inequality − including skin color, ethnicity, socioeconomic position, age, and territorial marginalization − and whether these were analyzed independently or through approaches capable of capturing compounded vulnerability.

### Ethical considerations

As this review analyzed existing literature and did not involve direct human contact, institutional ethical approval was not required.

## Results

The characteristics of the included studies are shown in Table 1. This scoping review included 82 studies, of which 65 addressed the social determinants of violence against women (Review Question 1) and 17 examined the health consequences of violence (Review Question 2).

**Table 1:** Summary of included studies by design, country, type of violence, and health outcomes (n = 82).

Characteristics	Review Question 1 (n = 65)	Review Question 2 (n = 17)
Study design		
Quantitative	56	13
Qualitative	9	3
Mixed	-	1
Country focus		
Brazil	30	6
Peru	9	4
Mexico	7	3
Chile	4	1
Colombia	2	-
Guatemala	2	-
Panama	2	-
Other/Underrepresented (with 1 study)	7	5
Regional/Multicountry	1	1
Type of violence studied		
Intimate partner violence	40	14
Dating violence	4	-
Sexual violence	5	4
Obstetric violence	2	-
Violence during pregnancy	-	6
General or unspecified violence against women	8	1
Violence with feminicide risk	-	1
Type of determinants		
Structural determinants only	8	-
Intermediary determinants only	11	6
Structural and intermediary	45	11
Health outcomes explored *		
Mental health (common mental disorders, depression, anxiety, post-traumatic stress disorders, suicidal ideation)	-	14
Maternal and perinatal health	-	6
Self-rated/Physiological health	-	4
Physiological/Biological outcomes (e.g. cortisol, IMT)	-	2
Health service utilization (prenatal care)	-	1

IMT: carotid artery intima-media thickness.

Note: categories of violence are not mutually exclusive, as several studies examined multiple forms of violence.

* Studies may contribute to more than one health outcome category.

### Geographical distribution and research concentration

The evidence base was geographically uneven. Brazil accounted for nearly half of all included studies (n = 36), followed by Peru (n = 13) and Mexico (n = 10). Only one study included multiple countries.

The literature was dominated by quantitative cross-sectional designs (n = 69). Qualitative studies constituted a smaller proportion (n = 12) of the evidence base.

### Forms of violence examined

As shown in Table 1, intimate partner violence was the most frequently examined form of violence across the included studies (n = 54). Sexual violence was analyzed either independently or in combination with intimate partner violence in a smaller subset of studies ^19^ ^,^ ^20^ ^,^ ^21^. Obstetric violence and institutional forms of violence were rarely addressed ^22^ ^,^ ^23^. Broader composite definitions of violence − including psychological, physical, sexual, economic, and symbolic forms − were employed in a minority of studies ^24^ ^,^ ^25^ ^,^ ^26^.

Fewer studies explicitly examined structural determinants, including income inequality, racialized exclusion, territorial segregation, and institutional arrangements ^22^ ^,^ ^27^ ^,^ ^28^ ^,^ ^29^.

Regarding health outcomes, the literature focused predominantly on mental health ^30^ ^,^ ^31^ ^,^ ^32^ ^,^ ^33^ ^,^ ^34^. Maternal and reproductive health outcomes were analyzed less frequently ^35^ ^,^ ^36^ ^,^ ^37^ ^,^ ^38^, whereas broader physical and cardiovascular outcomes were rare ^21^ ^,^ ^39^.

### Prevalence of violence against women

The reported prevalence of violence against women varied substantially across countries, age groups, and operational definitions, reflecting both contextual heterogeneity and methodological differences in measurement.

Among adolescents, dating violence was highly prevalent, particularly emotional and controlling behaviors. In urban public secondary schools in Panama, dating violence was highly prevalent among girls, with emotional violence being the most frequently reported form (61.6%) ^40^.

Among adult women, lifetime intimate partner violence prevalence ranged widely across settings. In Bolivia, nearly half of women reported experiencing some form of intimate partner violence ^31^, with psychological violence predominating. Similar patterns were observed in Mexico and Peru ^19^ ^,^ ^28^ ^,^ ^41^. Lower prevalence estimates were reported in Guatemala (13.9%) and Honduras (16.45%) ^37^ ^,^ ^42^.

In Brazil, prevalence estimates varied markedly by setting. National surveys reported past-year IPV prevalence below 10% ^29^ ^,^ ^43^, whereas rural or context-specific studies documented substantially higher rates. In rural Brazil, overall violence prevalence reached 18.6%, with psychological violence affecting 18%, physical violence 4.37%, and sexual violence 1.42% ^44^. Sexual violence prevalence similarly ranged widely, from approximately 9% among adolescents in Peru ^45^ to over 25% lifetime prevalence among Brazilian women ^46^.

### Review Question 1: social determinants of violence against women in Latin America and the Caribbean

#### Structural determinants of violence against women

#### a) Gender norms and cultural legitimization of violence

Across the literature, gender norms emerged as a central structural determinant of violence against women. Patriarchal norms, male dominance, and the social acceptance of control within intimate relationships were consistently associated with increased risks of emotional, physical, and sexual violence ^47^ ^,^ ^48^ ^,^ ^49^ ^,^ ^50^ ^,^ ^51^. Controlling behaviors − such as surveillance, jealousy, and restrictions on women’s autonomy − were frequently normalized across adolescent and adult relationships ^35^ ^,^ ^40^ ^,^ ^52^, often operating intergenerationally through socialization processes that framed violence as an expected component of marital or familial life ^26^ ^,^ ^53^ ^,^ ^54^.

At household and community levels, violence-supportive norms were socially embedded and reproduced within relational networks rather than confined to individual attitudes ^25^ ^,^ ^48^ ^,^ ^55^. In contexts shaped by migration and economic dependence, patriarchal community dynamics intensified symbolic, psychological, and economic violence, particularly where institutional support was limited ^25^ ^,^ ^48^, reinforcing everyday practices through which violence was tolerated and justified ^47^ ^,^ ^48^ ^,^ ^49^ ^,^ ^55^.

Within this normative landscape, adolescent dating violence was closely linked to jealousy, control, and romantic possession. Emotional and controlling behaviors − such as partner monitoring, checking phones or social media, and restricting interactions − were the most frequently reported forms across contexts ^40^ ^,^ ^49^ ^,^ ^50^. Despite their high prevalence, these behaviors were often minimized or normalized, whereas physical and sexual violence were more readily recognized as violence ^40^ ^,^ ^50^.

#### b) Education and symbolic power

Educational attainment functioned as a structural determinant with heterogeneous effects. In multiple contexts, higher levels of women’s education were associated with a reduced risk of physical and psychological intimate partner violence ^19^ ^,^ ^24^ ^,^ ^28^ ^,^ ^31^ ^,^ ^51^ ^,^ ^56^ ^,^ ^57^ ^,^ ^58^ ^,^ ^59^. Protective associations were particularly evident among Indigenous women in Mexico and Peru, where education was linked to increased awareness of rights and greater access to institutional and economic resources ^19^ ^,^ ^41^.

However, several studies reported elevated intimate partner violence risk when women’s educational attainment exceeded that of their partners. In Brazil and Honduras, women with higher education than their male partners had a higher risk of intimate partner violence ^60^ ^,^ ^61^, suggesting that relative educational differentials within couples may destabilize established gendered power dynamics.

#### c) Economic inequality, poverty, and structural vulnerability

Macroeconomic inequality was consistently associated with violence against women. Ecological analyses showed that countries and regions with higher income inequality − measured by the Gini coefficient or income ratios − had significantly higher intimate partner violence prevalence ^62^ ^,^ ^63^ ^,^ ^64^.

At household and community levels, poverty operated through multiple mechanisms, including economic stress, material deprivation, dependence, and reduced bargaining power ^43^ ^,^ ^46^ ^,^ ^65^ ^,^ ^66^. In Brazil, low socioeconomic status was associated with increased intimate partner violence risk, partly mediated by diminished social support and constrained living conditions ^27^ ^,^ ^65^ ^,^ ^66^. Food insecurity during pregnancy was strongly associated with intimate partner violence in Guatemala ^67^.

In Peru, women classified within middle wealth categories also exhibited elevated intimate partner violence risk compared with those in the highest wealth strata, indicating non-linear associations between economic position and violence exposure ^41^.

#### d) Women’s economic empowerment and backlash effects

Women’s economic empowerment emerged as a context-dependent structural determinant with both protective and risk-enhancing associations. In rural Guatemala, access to microfinance was associated with reductions in economic and emotional violence ^68^. In Mexico and Peru, greater decision-making autonomy was linked to lower intimate partner violence risk ^51^ ^,^ ^69^.

However, other studies documented increased intimate partner violence risk in contexts where shifts in women’s economic roles altered established household dynamics. In Brazil and Mexico, women’s employment or participation in conditional cash transfer programs reduced poverty but was associated, in certain contexts, with increased psychological intimate partner violence ^70^ ^,^ ^71^. Similarly, in Guatemala, employed women reported higher intimate partner violence risk despite the protective effects associated with decision-making autonomy ^60^.

#### e) Intersectionality of violence against women: skin color, space, and social exclusion

Evidence examining violence through racial, ethnic, and territorial lenses documented patterned and stratified inequalities across the region. In Brazil, Black and Mixed-race women − particularly those aged 18-24 − experienced disproportionately high levels of physical violence, frequently intersecting with unemployment, informal labor, lack of stable partnerships, and urban residence ^43^ ^,^ ^46^ ^,^ ^59^ ^,^ ^61^.

Similar patterns of compounded vulnerability were identified among Indigenous women in Mexico, Guatemala, and Ecuador, where elevated risks of intimate partner and sexual violence were associated with structural racism, geographic isolation, and limited access to education and services ^19^ ^,^ ^24^ ^,^ ^42^ ^,^ ^59^.

Spatial segregation consistently structured violence exposure across settings. Intimate partner violence was spatially clustered in poor urban territories and neighborhoods in Brazil and Colombia ^27^ ^,^ ^43^ ^,^ ^44^ ^,^ ^72^ ^,^ ^73^ ^,^ ^74^ ^,^ ^75^, with comparable clustering patterns documented across Brazilian cities ^27^ ^,^ ^72^.

In Peru, residential socioeconomic segregation was associated with increased physical intimate partner violence risk, operating through opposing mediation pathways: reinforcement of unfavorable gender norms increased risk, whereas social capital exerted protective effects ^76^.

Sexual violence followed similar socioeconomic and racial gradients. In Brazil and Chile, women from lower socioeconomic strata reported higher sexual victimization ^46^ ^,^ ^77^ ^,^ ^78^. Black women in Brazil experienced heightened vulnerability to sexual violence alongside poorer mental health outcomes ^20^ ^,^ ^46^ ^,^ ^72^.

Institutional inequalities were also evident in obstetric violence: younger, poorer, and less educated women relying on public healthcare systems in Brazil faced higher risks of mistreatment during childbirth ^22^, and in Mexico, approximately one-third of women reported obstetric violence, with public facilities and urban residence identified as key risk factors ^23^.

#### Intermediary social determinants of violence against women

#### a) Life-course timing and age

Age was associated with intimate partner violence risk in various ways across the life course. Younger women consistently exhibited higher vulnerability to intimate partner violence, frequently attributed to reduced autonomy, economic dependence, and relational instability ^24^ ^,^ ^41^ ^,^ ^43^ ^,^ ^62^ ^,^ ^79^ ^,^ ^80^.

Older women, particularly those with chronic illness or functional limitations, experienced increased psychological, economic, and physical violence, often within long-term relationships ^26^ ^,^ ^54^ ^,^ ^78^ ^,^ ^81^ ^,^ ^82^.

#### b) Neighborhood and community context

Neighborhood environments were associated with violence exposure through material, social, and spatial mechanisms. Intimate partner violence and child maltreatment co-occurred, sharing risks including neighborhood violence, absence of the biological father, and maternal depression ^74^. While aggregated neighborhood crime rates were not independently associated with intimate partner violence ^27^, local interpersonal dynamics − such as male-to-male aggression − were strongly associated with increased risk.

Neighborhood social capital demonstrated protective associations across settings, although certain network configurations and economic dependence were associated with increased sexual violence risk, varying by social and institutional context ^77^. Women residing in deprived urban neighborhoods experienced increased intimate partner violence and overlapping exposure to community and family violence ^72^ ^,^ ^74^ ^,^ ^75^. In Peru, environmental stressors, such as inadequate waste management and drought shocks, amplified intimate partner violence risks ^57^ ^,^ ^83^.

#### c) Employment precarity

Employment conditions constituted an important intermediary determinant. Precarious or informal employment increased intimate partner violence vulnerability, often by reinforcing economic dependence and instability ^75^. Participation in community economic activity was likewise associated with heightened risk in certain settings ^62^. In Mexico, stable employment posed greater intimate partner violence risk in rural areas than in urban settings, with women employed in the previous year reporting higher intimate partner violence prevalence ^70^.

#### d) Relational dynamics and partner behaviors

Relationship status was a consistent intermediary determinant. Cohabiting, non-married, or separated women reported higher levels of psychological intimate partner violence compared with married women ^25^ ^,^ ^31^ ^,^ ^41^ ^,^ ^58^ ^,^ ^62^ ^,^ ^78^ ^,^ ^79^ ^,^ ^84^.

Male partner behaviors − especially substance use − were among the factors most consistently associated with intimate partner violence. Alcohol consumption was repeatedly associated with physical, psychological, and sexual intimate partner violence across Brazil, Mexico, Peru, and Bolivia ^19^ ^,^ ^27^ ^,^ ^28^ ^,^ ^29^ ^,^ ^31^ ^,^ ^61^ ^,^ ^71^ ^,^ ^78^ ^,^ ^85^ ^,^ ^86^ ^,^ ^87^ ^,^ ^88^ ^,^ ^89^. Illicit drug use was also associated with greater intimate partner violence severity ^29^ ^,^ ^80^ ^,^ ^89^.

#### e) Women’s alcohol use

Alcohol use and alcohol use disorders were consistently associated with exposure to multiple forms of violence. In rural Brazil, lifetime psychological intimate partner violence was strongly associated with depression, recent alcohol use, and non-partnered status ^90^. Lifetime rape victimization was positively associated with alcohol use disorder and binge drinking, with lower prevalence observed among women with higher education and those who were married ^20^.

Past-year alcohol consumption was associated with a higher risk of intrafamilial and extrafamilial sexual assault, with patterns varying by sex and type of violence, including elevated extrafamilial rape among females ^45^. Similarly, lifetime sexual abuse was strongly associated with heavy drinking and former drinking status ^72^.

#### f) Migration

Migration influenced intimate partner violence dynamics through multiple mechanisms. Rural-to-urban migration in Peru increased women’s exposure to physical and sexual violence ^91^, while immigrant women in Chile faced heightened intimate partner violence risk due to social isolation and labor precarity ^87^. In transnational Mexican communities, remittance control and in-law surveillance reinforced symbolic and economic forms of violence ^25^.

#### g) Individual social capital

Social capital emerged as a key protective intermediary determinant against physical and psychological intimate partner violence. Strong social networks, especially neighborly support, were associated with lower intimate partner violence rates ^35^ ^,^ ^48^ ^,^ ^55^ ^,^ ^65^ ^,^ ^66^ ^,^ ^76^ ^,^ ^77^. However, its protective effects were attenuated in contexts characterized by entrenched gender norms, residential segregation, and institutional neglect ^76^.

#### h) Childhood exposure to parental violence: a pathway to intimate partner violence in adulthood

Childhood exposure to parental violence, including witnessing aggression, was a significant predictor of intimate partner violence in adulthood, reinforcing intergenerational cycles of violence ^19^ ^,^ ^28^ ^,^ ^41^ ^,^ ^53^ ^,^ ^57^ ^,^ ^78^ ^,^ ^84^ ^,^ ^85^ ^,^ ^86^ ^,^ ^87^ ^,^ ^89^ ^,^ ^92^. Perpetrators often replicated abusive behaviors observed in their families, underscoring the persistence of violence across generations ^53^ ^,^ ^61^.

#### i) Severity, recurrence, and lethality in pregnancy-related intimate partner violence

Administrative data further characterized severity patterns of reported intimate partner violence. Notifications were predominantly physical and more frequent among women aged 20-39, during pregnancy, when violence occurred at home, in recurrent episodes, and when aggressor alcohol use was reported ^88^.

Among pregnant women with recorded violence, lethality risk varied by assault characteristics. Death was strongly associated with firearm and sharp-object assaults, whereas higher education, being married or stably partnered, and residence in larger municipalities were associated with lower mortality risk ^93^.

### Review Question 2: health consequences of violence against women in Latin America and the Caribbean

#### Mental health consequences of violence against women

Across Latin America and the Caribbean, exposure to violence against women − particularly intimate partner violence − was consistently associated with a wide spectrum of adverse mental health outcomes, including depression, anxiety, post-traumatic stress symptoms, suicidal ideation, and common mental disorders ^31^ ^,^ ^32^ ^,^ ^33^ ^,^ ^42^ ^,^ ^94^ ^,^ ^95^. Evidence converged on a clear gradient: greater severity, multiplicity, and chronicity of violence were associated with progressively worse psychological outcomes.

Population-based studies in Brazil, Bolivia, and El Salvador documented markedly higher prevalence of depression, anxiety, and common mental disorders (CMD) among women exposed to intimate partner violence compared with non-exposed women ^30^ ^,^ ^33^ ^,^ ^96^. Psychological violence exerted mental health effects comparable to physical violence, while sexual intimate partner violence was consistently associated with the most severe outcomes, particularly when co-occurring with other forms of abuse ^31^ ^,^ ^33^. Polyvictimization yielded the highest predicted probabilities of depression and severe psychological distress ^31^ ^,^ ^33^.

Evidence further supported temporal associations, with recent and sustained intimate partner violence exposure increasing the postpartum incidence of CMD ^33^ ^,^ ^97^, while childhood sexual abuse independently amplified CMD risk in adulthood ^32^, indicating cumulative and life-course effects.

During the COVID-19 pandemic in Brazil ^98^, high levels of recent intimate partner violence coincided with elevated prevalence of depression and suicidal ideation. A dose-response relationship was observed, whereby exposure to multiple forms of violence was associated with substantially worse mental health outcomes. Structural stressors, including food insecurity and racialized disadvantage, were linked to intensified psychological harm, whereas co-residence with children was associated with lower depressive symptomatology.

In Mexico, high-control intimate partner violence − characterized by coercive and surveillance behaviors − was more strongly associated with depression, anxiety, and suicidality than moderate-control intimate partner violence ^42^.

Qualitative evidence added contextual depth to these quantitative findings. Among Indigenous Maya women in rural Guatemala, profound emotional distress, somatic symptoms, and suicidal ideation were documented alongside extremely low levels of treatment seeking ^94^. In Chile, women attending specialized support centers reported a high prevalence of psychological distress, post-traumatic stress disorder (PTSD), and moderate-to-severe depression ^34^. In Peru, exposure to violence involving feminicide risk was associated with substantial increases in depression and substance use ^99^.

#### Maternal and perinatal health outcomes

Violence during pregnancy was associated with adverse maternal health outcomes and disrupted engagement with health services. In Guyana, intimate partner violence during pregnancy was strongly associated with suicidal ideation and poorer self-rated health ^96^. In Brazil, physical intimate partner violence was associated with insufficient gestational weight gain, a clinically relevant indicator linked to adverse maternal and neonatal outcomes ^36^. Cohort evidence further showed that psychological and physical violence during pregnancy was associated with increased depressive symptoms both during the prenatal period and postpartum, with socioeconomic disadvantage and limited social support contributing to these associations ^35^.

Physiological evidence from Peru indicated that childhood abuse − rather than intimate partner violence alone − was associated with elevated hair cortisol and cortisone concentrations during pregnancy ^38^, suggesting long-term biological embedding of early trauma.

Service utilization was also affected. In Honduras, exposure to physical and sexual intimate partner violence was associated with increased likelihood of insufficient prenatal care attendance, even when care was initiated early ^37^.

#### Physical and self-rated health outcomes

Exposure to violence was associated with broader physical health consequences. In Mexico, lifetime exposure to physical and sexual violence − particularly violence perpetrated by strangers − was associated with increased carotid intima-media thickness and higher odds of subclinical atherosclerosis ^25^.

In Brazil, both family and community violence were associated with poorer self-rated health, with the strongest effects observed among individuals exposed to multiple forms of violence ^39^.

## Discussion

This scoping review critically analyzes how structural and intermediary SDH have been conceptualized, operationalized, and examined in relation to violence against women in Latin America and the Caribbean, and their implications for physical and mental health outcomes. By explicitly organizing the evidence through the SDH and applying an intersectional interpretive lens when possible, the review advances a structurally grounded synthesis that clarifies what is known, what remains under-theorized, and where policy-relevant evidence remains limited.

### Overview of the findings

As synthesized in Figure 2, violence against women in Latin America and the Caribbean is structurally produced and socially patterned rather than an isolated interpersonal event, embedded within systems of socioeconomic stratification, gendered norms, racialized hierarchies, and institutional power ^25^ ^,^ ^26^ ^,^ ^35^ ^,^ ^40^ ^,^ ^47^ ^,^ ^48^ ^,^ ^49^ ^,^ ^50^ ^,^ ^51^ ^,^ ^52^ ^,^ ^53^ ^,^ ^54^ ^,^ ^55^. Although intimate partner violence predominates, sexual, psychological, economic, and obstetric violence are also documented, the latter revealing the institutional reproduction of harm ^22^ ^,^ ^23^.

**Figure 2:**
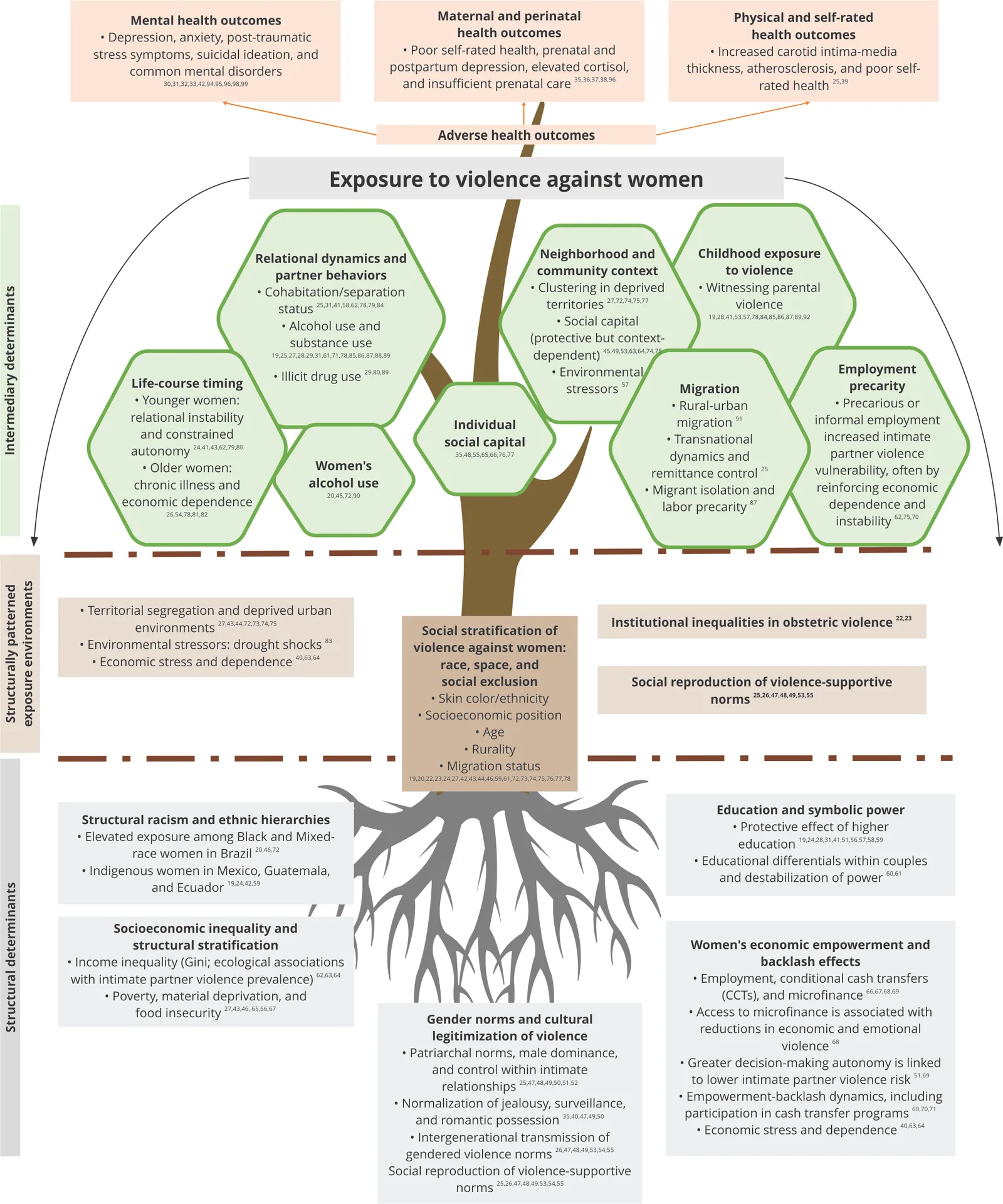
Social determinants of violence against women in Latin America and the Caribbean: a structurally grounded framework.

Economic inequality emerged as a central structural determinant. Ecological evidence linked higher income inequality to higher intimate partner violence prevalence ^62^ ^,^ ^63^ ^,^ ^64^. This inequality, together with household deprivation, is consistently associated with increased intimate partner violence risk ^43^ ^,^ ^65^ ^,^ ^66^. At the same time, women’s education and economic autonomy may reduce dependence while heightening risk where patriarchal norms are challenged ^60^ ^,^ ^61^. Racialized and territorial inequalities further stratify exposure, with clustering of violence in marginalized urban areas and a disproportionate impact on Black and Indigenous women ^27^ ^,^ ^43^ ^,^ ^44^ ^,^ ^72^ ^,^ ^73^ ^,^ ^74^ ^,^ ^75^ ^,^ ^76^ ^,^ ^77^. Obstetric violence illustrates institutional inequities affecting younger, poorer, and less educated women reliant on public systems ^22^ ^,^ ^23^.

Intermediary determinants mediate these structural conditions across the life course. Younger women face heightened vulnerability linked to constrained autonomy ^24^ ^,^ ^41^ ^,^ ^43^ ^,^ ^62^ ^,^ ^79^ ^,^ ^80^, while older women remain exposed to psychological and economic abuse ^26^ ^,^ ^54^ ^,^ ^78^ ^,^ ^81^ ^,^ ^82^. Neighborhood stressors increase risk, whereas social capital may buffer it ^27^ ^,^ ^72^ ^,^ ^74^ ^,^ ^75^ ^,^ ^77^. Partner alcohol and substance use were consistently associated with greater severity and recurrence of violence ^19^ ^,^ ^27^ ^,^ ^28^ ^,^ ^29^ ^,^ ^31^ ^,^ ^61^ ^,^ ^71^ ^,^ ^78^ ^,^ ^85^ ^,^ ^86^ ^,^ ^87^ ^,^ ^88^ ^,^ ^89^.

Violence is associated with cumulative mental, maternal, and somatic health consequences, including depression, anxiety, post-traumatic stress, suicidal ideation, adverse pregnancy outcomes, reduced prenatal care, and cardiovascular risk ^25^ ^,^ ^31^ ^,^ ^32^ ^,^ ^33^ ^,^ ^35^ ^,^ ^36^ ^,^ ^37^ ^,^ ^38^ ^,^ ^39^ ^,^ ^42^ ^,^ ^94^ ^,^ ^95^ ^,^ ^96^. Together, these findings position violence against women in Latin America and the Caribbean as part of a continuum of social and health inequality embodied across the life course ^8^ ^,^ ^9^.

### Research gaps and future research directions

#### Underexplored forms of violence beyond intimate partner violence

Despite the breadth of the literature reviewed, research in Latin America and the Caribbean remains disproportionately concentrated on intimate partner violence, with comparatively limited attention to other pervasive and structurally mediated forms of violence. Obstetric violence, economic violence, workplace harassment, and symbolic or institutional violence remain under-theorized and empirically underexplored, despite their clear implications for women’s autonomy, dignity, and health.

Future research should therefore expand beyond interpersonal intimate partner violence to examine how health and legal systems, social norms, and organizational practices sustain violence, including in maternal and reproductive healthcare. Comparative designs across different forms of violence − interpersonal, institutional, and community-based − would help identify shared structural determinants and violence-specific mechanisms, strengthening evidence for integrated prevention strategies.

#### Violence as a continuum across the life course and social spaces

The review supports a continuum perspective, whereby violence emerges as cumulative and evolving across time and social settings. However, explicit life-course designs and analyses of temporal sequencing remain limited across the regional evidence base.

Future research should prioritize longitudinal and cohort designs that can trace how early-life exposure, structural disadvantage, and repeated victimization interact to shape long-term health trajectories. Integrating spatial approaches would further clarify how territorial deprivation and segregation shape exposure to violence and health consequences over time.

#### Intersectionality as an analytical framework, not a descriptive attribute

Although various studies documented higher risks of violence among Black, Indigenous, rural, and socioeconomically disadvantaged women, intersectionality was rarely operationalized analytically. Future research should explicitly adopt intersectional methodologies that move beyond additive modeling, including stratified analyses, interaction terms, and mixed-methods designs that connect lived experience to structural positioning. This is particularly urgent in rural and Indigenous contexts, where structural racism, geographic isolation, and institutional neglect emerged as recurring features shaping violence and barriers to protection.

#### Structural determinants and political economy of violence

A persistent gap concerns the limited integration of macro-structural determinants into empirical models and explanations of violence. Future research should strengthen the integration of political economy perspectives with gender and SDH frameworks, explicitly examining how labor market segmentation, economic crises, austerity, and social protection regimes shape women’s exposure to violence. Particular attention should be paid to policy-mediated dynamics of empowerment and backlash, including conditional cash transfer programs and labor participation, which may reduce deprivation while generating new forms of risk in patriarchal contexts.

#### Emerging determinants: climate change and digital technologies

Future research should systematically investigate how climate-related stressors, environmental degradation, and climate-induced mobility or immobility interact with existing SDH to shape violence risk, particularly in rural and resource-dependent contexts. Similarly, research should examine the role of digital technologies in expanding coercive control and surveillance, while also assessing potential protective uses for support and help-seeking, especially for adolescents and young women.

#### Toward holistic policy evaluation and knowledge translation

Future studies should prioritize rigorous evaluation of multisectoral policies and programs, including economic, health, education, and social protection interventions. Embedding evaluation within real-world implementation and involving affected communities in co-producing knowledge will be essential for translating evidence into equitable and effective public health action.

#### Policy implications

The findings of this scoping review indicate that policies addressing violence against women in Latin America and the Caribbean must move beyond individualized and reactive approaches and engage directly with the structural and intermediary social determinants that sustain violence. Strategies focused exclusively on criminal justice or individual behavior change are insufficient in contexts where gender norms, socioeconomic inequality, racialized exclusion, and territorial marginalization systematically shape women’s exposure to violence.

At the structural level, policies aimed at reducing poverty, educational inequalities, labor precarity, and gendered economic dependence are central to violence prevention. Economic empowerment initiatives, including cash transfers and microfinance, should be accompanied by gender-transformative components that address patriarchal backlash and renegotiation of power within households. Education policies should integrate critical gender and rights-based curricula from early adolescence, particularly in public and rural education systems where vulnerabilities to dating and intimate partner violence are concentrated.

At the intermediary level, strengthening social protection systems, community-based social capital, and access to culturally competent health, legal, and psychosocial services is essential. Health systems represent a critical entry point for prevention and early response, particularly during pregnancy and childbirth; addressing obstetric violence requires institutional accountability across health and justice systems, professional training, and monitoring mechanisms that prioritize dignity, informed consent, and equity in public healthcare services. Integrating routine, ethically grounded screening for violence within primary care and reproductive health services must be accompanied by referral pathways that are accessible to Indigenous, rural, and migrant women.

Finally, policy responses must explicitly incorporate intersectional and territorial perspectives. Programs designed without attention to skin color, ethnicity, migration status, rurality, and life-course exposure risk reproduce existing inequities. Emerging challenges − including climate-related stressors and digital forms of coercion − should be proactively incorporated into violence prevention frameworks. Sustained investment in data systems, mixed-methods research, and community-led monitoring is necessary to inform adaptive, context-sensitive policies capable of addressing the evolving continuum of violence against women in the region.

### Limitations

This review is constrained by uneven geographical representation, with a concentration of studies in a limited number of countries. Underrepresentation of rural, Indigenous, Afro-descendant, and migrant women reflects broader inequities in research production. The reliance on peer-reviewed literature may have excluded relevant gray literature, particularly regarding institutional and community-level dynamics. Heterogeneity in study design and measurement, combined with the predominance of cross-sectional analyses, limits causal inference and comparability. Finally, although interpreted through an intersectional lens, few primary studies formally operationalized intersectionality, restricting the synthesis of compounded disadvantage.

## Conclusions

Violence against women in Latin America and the Caribbean is structurally embedded, socially patterned, and cumulatively embodied across the life course. Exposure is shaped by intersecting systems of gender inequality, racialized hierarchies, economic stratification, and territorial marginalization, while intermediary determinants mediate risk and resilience. Health consequences extend beyond psychological distress to maternal, physiological, and long-term somatic outcomes.

Addressing violence against women in the region requires multisectoral, equity-oriented strategies that integrate political economy, gender-transformative, and territorial perspectives. Advancing the evidence base demands longitudinal designs, intersectional methodologies, and stronger integration of structural determinants into empirical modeling. Without confronting the social arrangements that produce violence, prevention efforts will remain partial and uneven.

## Data Availability

The sources of information used in the study are indicated in the body of the article.

## References

[1] World Health Organization Gender based violence is a public health issue: using a health systems approach..

[2] Organización Panamericana de la Salud Violencia contra la mujer..

[3] World Health Organization Violencia contra las mujeres: estimaciones para 2018. Estimaciones mundiales, regionales y nacionales de la prevalencia de la violencia de pareja contra las mujeres y estimaciones mundiales y regionales de la prevalencia de la violencia sexual fuera de la pareja contra las mujeres..

[4] Coll CV, Ewerling F, García-Moreno C, Hellwig F, Barros AJ (2020). Intimate partner violence in 46 low-income and middle-income countries an appraisal of the most vulnerable groups of women using national health surveys. BMJ Global Health.

[5] Office of the High Commissioner for Human Rights, United Nations Declaration on the Elimination of Violence against Women..

[6] Organización de las Naciones Unidas Mujeres La pandemia en la sombra: violencia contra las mujeres durante el confinamiento..

[7] Eapen DJ, Tsusaki RB, Mullassery DG, Goolsby C, Lee K, Wardell DW (2023). A systematic review of women's experiences of interpersonal violence during the COVID-19 pandemic. Nurs Womens Health.

[8] Solar O, Irwin A (2010). A conceptual framework for action on the social determinants of health..

[9] Comisión de la Organización Panamericana de la Salud sobre Equidad y Desigualdades en Salud en las Américas (2019). Sociedades justas: equidad en la salud y vida digna..

[10] Caudillo-Ortega L, Hernández-Ramos MT, Flores-Arias ML (2017). Análisis de los determinantes sociales de la violencia de género. Ra Ximhai.

[11] Langer A, Meleis A, Knaul FM, Atun R, Aran M, Arreola-Ornelas H (2015). Women and health the key for sustainable development. Lancet.

[12] Mannell J, Lowe H, Brown L, Mukerji R, Devakumar D, Gram L (2022). Risk factors for violence against women in high-prevalence settings a mixed-methods systematic review and meta-synthesis. BMJ Glob Health.

[13] Yakubovich AR, Stöckl H, Murray J, Melendez-Torres GJ, Steinert JI, Glavin CEY (2018). Risk and protective factors for intimate partner violence against women systematic review and meta-analyses of prospective-longitudinal studies. Am J Public Health.

[14] Stubbs A, Szoeke C (2022). The effect of intimate partner violence on the physical health and health-related behaviors of women a systematic review of the literature. Trauma Violence Abuse.

[15] Loraine JB, Meghna R, Charlotte W, Karen D (2018). Recent intimate partner violence against women and health a systematic review and meta-analysis of cohort studies. BMJ Open.

[16] Collins P (2019). Intersectionality as critical social theory.

[17] Armstrong R, Hall BJ, Doyle J, Waters E (2011). 'Scoping the scope' of a cochrane review.. J Public Health (Oxf).

[18] Peters MD, Godfrey CM, Khalil H, McInerney P, Parker D, Soares CB (2015). Guidance for conducting systematic scoping reviews. J Int J Evid Based Healthc.

[19] Valdez-Santiago R, Híjar M, Rojas Martínez R, Avila Burgos L, Arenas Monreal ML (2013). Prevalence and severity of intimate partner violence in women living in eight indigenous regions of Mexico. Soc Sci Med.

[20] Massaro LTS, Adesse L, Laranjeira R, Caetano R, Madruga CS (2019). Rape in Brazil and relationships with alcohol consumption estimates based on confidential self-reports. Cad Saúde Pública.

[21] Flores-Torres MH, Lynch R, Lopez-Ridaura R, Yunes E, Monge A, Ortiz-Panozo E (2017). Exposure to violence and carotid artery intima-media thickness in Mexican women. J Am Heart Assoc.

[22] Mesenburg M, Victora C, Jacob Serruya S, Ponce de León R, Damaso A, Domingues M (2018). Disrespect and abuse of women during the process of childbirth in the 2015 Pelotas birth cohort.. Reprod Health.

[23] Castro R, Frías S (2020). Obstetric violence in Mexico results from a 2016 national household survey. Violence Against Women.

[24] Edeby A, San Sebastián M (2021). Prevalence and sociogeographical inequalities of violence against women in Ecuador a cross-sectional study. Int J Equity Health.

[25] Flores YYR (2016). La violencia contra mujeres en comunidades transnacionales de San Luis Potosí, México un problema de salud pública. Cad Saúde Pública.

[26] Queiroga Souto R, Barbosa Merighi M, Guruge S, Jesus MP (2021). Factors associated with the risk of violence against older adult women a cross-sectional study. Rev Latinoam Enferm.

[27] Kiss L, Schraiber L, Hossain M, Watts C, Zimmerman C (2015). The link between community-based violence and intimate partner violence the effect of crime and male aggression on intimate partner violence against women. Prev Sci.

[28] Safranoff A (2017). Violencia psicológica hacia la mujer ¿cuáles son los factores que aumentan el riesgo de que exista esta forma de maltrato en la pareja?. Salud Colect.

[29] Ally E, Laranjeira R, Viana M, Pinsky I, Caetano R, Mitsuhiro S (2016). Intimate partner violence trends in Brazil data from two waves of the Brazilian National Alcohol and Drugs Survey. Braz J Psychiatry.

[30] Avanci J, Assis S, Oliveira R (2013). A cross-sectional analysis of women's mental health problems examining the association with different types of violence among a sample of Brazilian mothers. BMC Women's Health.

[31] Meekers D, Pallin S, Hutchinson P (2013). Intimate partner violence and mental health in Bolivia. BMC Women's Health.

[32] Navarro-Mantas L, Lemus S, Megías J (2021). Mental health consequences of intimate partner violence against women in El Salvador. Violence Against Women.

[33] Mendonça MFS, Ludermir AB (2017). Intimate partner violence and incidence of common mental disorder. Rev Saúde Pública.

[34] Labra-Valerdi P, Chacón-Moscoso S, Sanduvete-Chaves S (2022). Predictive factors of mental health in survivors of intimate partner violence in Chile. J Interpers Violence.

[35] Ribeiro MRC, Silva AAM, Schraiber LB, Murray J, Alves MTSSB, Batista RFL (2020). Inversion of traditional gender roles and intimate partner violence against pregnant women. Cad Saúde Pública.

[36] Batista Flor R, Marques ES, Oliveira AS, Hasselmann MH (2023). Intimate partner physical violence and inadequate weight gain in pregnancy. Rev Bras Saúde Matern Infant.

[37] Sebert Kuhlmann A, Foggia J, Fu Q, Sierra M (2017). Intimate partner violence as a predictor of antenatal care service utilization in Honduras. Rev Panam Salud Pública.

[38] Kelsall NC, Sanchez SE, Rondon MB, Valeri L, Juvinao-Quintero D, Kirschbaum C (2023). Association between trauma exposure and glucocorticosteroid concentration in hair during pregnancy. Psychoneuroendocrinology.

[39] Andrade AB, Azeredo CM, Peres MFT (2020). Exposure to community and family violence and self-rated health in the Brazilian population. Rev Bras Epidemiol.

[40] Gabster A, Xavier Hall C, Yu Pon A, Millender E, Wong F, Pascale J (2022). Dating violence prevalence and risk factors among adolescents (14-19 years) in urban public schools in Panama. Lancet Reg Health Am.

[41] Burgos-Muñoz R, Soriano-Moreno A, Bendezu-Quispe G, Urrunaga-Pastor D, Toro-Huamanchumo C, Benites-Zapata V (2021). Intimate partner violence against reproductive-age women and associated factors in Peru evidence from national surveys, 2015-2017. Heliyon.

[42] Aguerrebere M, Frías S, Smith Fawzi M, López R, Raviola G (2021). Intimate partner violence types and symptoms of common mental disorders in a rural community of Chiapas, Mexico implications for global mental-health practice. PLOS One.

[43] Castro-Alves J, Bastos FI, Cobo B, De Boni RB (2023). Physical violence against women in Brazil findings from the 3rd Brazilian household survey on substance use. Glob Public Health.

[44] Stochero L, Pinto LW (2024). Prevalence of violence against women living in rural areas and associated factors a cross-sectional study based on the 2019 National Health Survey. Ciênc Saúde Colet.

[45] Valle R, Bernabé-Ortiz A, Gálvez-Buccollini J, Gutiérrez C, Martins S (2018). Intrafamilial and extrafamilial sexual assault and its association with alcohol consumption. Rev Saúde Pública.

[46] da Silva J, Roncalli A (2018). Prevalence of sexual violence in Brazil associated individual and contextual factors. Int J Public Health.

[47] Rodney R, Gastaldo D, Trotz DA, Crooks CV (2021). Sex as boys' fame, but girls' shame adversarial adolescent gender roles and gender-based violence in Guyana. J Interpers Violence.

[48] Lennon S, Aramburo A, Garzón E, Arboleda M, Fandiño-Losada A, Pacichana-Quinayaz S (2021). A qualitative study on factors associated with intimate partner violence in Colombia. Ciênc Saúde Colet.

[49] Sanhueza T, Lalande C, Lessard G (2022). A gender study of the social representations of dating violence in Chilean adolescents. SN Soc Sci.

[50] Campeiz AB, Carlos DM, Campeiz AF, Silva JLD, Freitas LA, Ferriani M (2020). Violence in intimate relationships from the point of view of adolescents perspectives of the Complexity Paradigm. Rev Esc Enferm USP.

[51] Cortés J, Rivera Aragón C, Amorin de Castro S, Filipa E, Rivera Rivera L (2015). Violencia de pareja en mujeres: prevalencia y factores asociados.. Acta de Investigación Psicológica.

[52] Terrazas-Carrillo E, McWhirter P (2015). Employment status and intimate partner violence among Mexican women. J Interpers Violence.

[53] Paixão G, Gomes N, Diniz N, Carvalho e Lira M, Carvalho M, da Silva R (2015). Women experiencing the intergenerationality of conjugal violence.. Rev Latinoam Enferm.

[54] Oliveira ASLA, Moreira LR, Meucci RD, Paludo SS (2021). Psychological violence against women practiced by intimate partners a cross-sectional study in a rural area of Rio Grande do Sul, Brazil, 2017. Epidemiol Serv Saúde.

[55] Shakya HB, Hughes DA, Stafford D, Christakis NA, Fowler JH, Silverman JG (2016). Intimate partner violence norms cluster within households an observational social network study in rural Honduras. BMC Public Health.

[56] Weitzman A (2018). Does increasing women's education reduce their risk of intimate partner violence Evidence from an education policy reform. Criminology.

[57] Fiestas F, Rojas R, Gushiken A, Gozzer E (2012). ¿Quién es la víctima y quién el agresor en la violencia física entre parejas?: estudio epidemiológico en siete ciudades del Perú.. Rev Peru Med Exp Salud Pública.

[58] Pinto I, Bernal R, Souza MF, Malta D (2021). Factors associated with death in women with intimate partner violence notification in Brazil. Ciênc Saúde Colet.

[59] Santamaría A, Gerald C, Chamorro F, Herrera V, Flores H, Sandoval I (2022). Social determinants of violence against women in Panama results from population-based cross-sectional studies and a femicide registry. Int Health.

[60] Meeker K, Hayes B, Randa R, Saunders J (2020). Examining risk factors of intimate partner violence victimization in Central America a snapshot of Guatemala and Honduras. Int J Offender Ther Comp Criminol.

[61] Vasconcelos NM, Andrade FMD, Gomes CS, Bernal RTI, Malta DC (2022). Physical violence against women by an intimate partner: analysis of VIVA Survey 2017.. Ciênc Saúde Colet.

[62] Torres Munguía J, Martínez-Zarzoso I (2022). Determinants of emotional intimate partner violence against women and girls with children in Mexican households an ecological framework. J Interpers Violence.

[63] Yapp E, Pickett KE (2019). Greater income inequality is associated with higher rates of intimate partner violence in Latin America. Public Health.

[64] Lopes Pereira M, Gaspar R (2021). Socioeconomic factors associated with reports of domestic violence in large Brazilian cities. Front Public Health.

[65] Ribeiro MR, Silva AA, Alves MT, Batista RF, Ribeiro CC, Schraiber LB (2017). Effects of socioeconomic status and social support on violence against pregnant women a structural equation modeling analysis. PLOS One.

[66] Costa DCS, Ribeiro MRC, Batista RFL, Valente CV, Ribeiro JVF, Almeida LA (2017). Factors associated with physical violence against pregnant women from São Luís, Maranhão State, Brazil an approach using structural equation modeling. Cad Saúde Pública.

[67] Lee AE, Rivera C, Montes SB, Jimenez-Zambrano A, Nacht A, Bolanos A (2021). Identifying prevalence and risk factors for intimate partner violence in pregnant women in rural Guatemala. J Womens Health Dev.

[68] Cepeda I, Lacalle-Calderon M, Torralba M (2021). Microfinance and violence against women in rural Guatemala. J Interpers Violence.

[69] Barón-Lozada F, Basualdo-Meléndez G, Vargas-Fernández R, Hernández-Vásquez A, Bendezu-Quispe G (2022). Women's autonomy and intimate partner violence in Peru analysis of a National Health Survey. Int J Environ Res Public Health.

[70] Canedo A, Morse S (2021). An estimation of the effect of women's employment on the prevalence of intimate partner violence in Mexico. J Interpers Violence.

[71] Leite T, Moraes C, Reichenheim M, Deslandes S, Salles-Costa R (2022). The role of income on the relationship between the Brazilian Cash Transfer Program (Programa Bolsa Família) and intimate partner violence evidence from a Multigroup Path Analysis. J Interpers Violence.

[72] Barbosa KGN, Walker BB, Schuurman N, Cavalcanti S, Ferreira EFE, Ferreira RC (2019). Epidemiological and spatial characteristics of interpersonal physical violence in a Brazilian city a comparative study of violent injury hotspots in familial versus non-familial settings, 2012-2014. PLOS One.

[73] Camargo Freile I, Flórez Lozano K, Sarmiento Crespo C, Vecchio Camargo C, Rodríguez Acosta S, Florez-Garcia V (2022). Risk of violence from a current or former partner associated factors and classification in a nationwide study in Colombia. PLOS One.

[74] Buffarini R, Coll CVN, Moffitt T, Freias da Silveira M, Barros F, Murray J (2021). Intimate partner violence against women and child maltreatment in a Brazilian birth cohort study: co-occurrence and shared risk factors.. BMJ Glob Health.

[75] Kim H, Grogan-Kaylor A, Han Y, Maurizi L, Delva J (2013). The association of neighborhood characteristics and domestic violence in Santiago, Chile. J Urban Health.

[76] Benavides M, León J, Etesse M, Espezúa L, Stuart J (2019). Exploring the association between segregation and physical intimate partner violence in Lima, Peru the mediating role of gender norms and social capital. SSM Popul Health.

[77] Ilabaca Baeza P, Gaete Fiscella JM, Hatibovic Díaz F, Roman Alonso H (2022). Social, economic and human capital risk or protective factors in sexual violence?. Int J Environ Res Public Health.

[78] Leite F, Amorim M, Wehrmeister F, Gigante D (2017). Violence against women, Espírito Santo, Brazil. Rev Saúde Pública.

[79] Signorelli MC, Souza FG, Pinheiro RVB, Valente J, Andreoni S, Rezende LFM (2023). Panorama of intimate partner violence against women in Brazil and its association with self-perception of health: findings from a national representative survey.. J Interpers Violence.

[80] Conceição HND, Coelho SF, Madeiro AP (2021). Prevalence and factors associated with intimate partner violence during pregnancy in Caxias, state of Maranhão, Brazil, 2019-2020. Epidemiol Serv Saúde.

[81] Souto RQ, Merighi MA, Guruge S, Jesus MC (2015). Older Brazilian women's experience of psychological domestic violence a social phenomenological study. Int J Equity Health.

[82] Oliveira AA, Lazarini CA, Marin MJS, Alarcon MFS, Moraes MAA, Higa EFR (2023). Violence against elderly women. Cogitare Enferm.

[83] Díaz J-J, Saldarriaga V (2023). A drop of love? Rainfall shocks and spousal abuse: evidence from rural Peru.. J Health Econ.

[84] Ruíz-Aquino M (2021). Predictores de la violencia ejercida contra la mujer en departamentos de alta prevalencia del Perú. Rev Cient Salud UNITEPC.

[85] Carvalho M, Oliveira JF, Gomes NP, Santos MM, Estrela FM, Duarte H (2018). Interface between conjugal violence and alcohol consumption by the partner. Rev Bras Enferm.

[86] Ludermir A, Araújo T, Valongueiro S, Muniz M, Silva E (2017). Previous experience of family violence and intimate partner violence in pregnancy. Rev Saúde Pública.

[87] Mella M, Binfa L, Carrasco A, Cornejo C, Cavada G, Pantoja L (2021). Violencia contra la mujer durante la gestación y postparto infligida por su pareja en Centros de Atención Primaria de la zona norte de Santiago, Chile. Rev Med Chile.

[88] Mascarenhas MDM, Tomaz GR, Meneses GMS, Rodrigues MTP, Pereira VOM, Corassa RB (2020). Analysis of notifications of intimate partner violence against women, Brazil, 2011-2017.. Rev Bras Epidemiol.

[89] Madruga C, Viana M, Abdalla R, Caetano R, Laranjeira R (2017). Pathways from witnessing parental violence during childhood to involvement in intimate partner violence in adult life the roles of depression and substance use. Drug Alcohol Rev.

[90] Oliveira J, Kerr-Correa F, Lima M, Bertolote J, Tucci A (2013). Sexual abuse and alcohol use among women in metropolitan São Paulo, Brazil a general population study. Rev Bras Epidemiol.

[91] Terrazas J, Blitchtein D (2022). Rural-urban migration as a factor associated with physical and sexual intimate partner violence Peru 2015-2017: a secondary analysis of a national study.. BMC Womens Health.

[92] Hernández-Vásquez A, Chévez-Ramírez T, Rojas-Roque C (2022). Intimate partner violence among women of reproductive age in Nicaragua results from a nationwide survey. J Interpers Violence.

[93] Soares MQ, Melo CM, Pinto IV, Bevilacqua PD (2023). Mortalidade de mulheres com notificação de violência durante a gravidez no Brasil um estudo caso-controle. Cad Saúde Pública.

[94] Rogg M, Pezzia C (2023). Victimization and distress in indigenous maya women a qualitative investigation of gender-based violence and mental health outcomes in rural Guatemala. J Fam Violence.

[95] Ortega Ceballos P, Rivera Rivera L, Reynales Shigematsu L, Austria Corrales F, Toledano-Toledano F, Pérez Amezcua B (2023). Psychological distress, intimate partner violence and substance use in a representative sample from Mexico a structural equation model. Front Public Health.

[96] Miller L, Contreras-Urbina M (2021). Exploring the determinants and outcomes of intimate partner violence during pregnancy for Guyanese women results from a nationally representative cross-sectional household survey. Rev Panam Salud Pública.

[97] Ribeiro SVO, Batista RFR, Pessoa KC, Simões VMF, Figuereido FP, Bettiol H (2019). Violence and depressive symptoms during pregnancy in BRISA cohort using structural equation modeling approach. Rev Bras Saúde Mater Infant.

[98] Baumont AC, Oliveira GS, Figueiredo JB, Dos Santos JF, Genro BP, Habigzang LF (2024). Intimate partner violence and women's mental health during the COVID-19 pandemic in Brazil. Trends Psychiatry Psychother.

[99] Hernández W (2021). Violence with femicide risk its effects on women and their children. J Interpers Violence.

