# Supplementary material for: Social determinants of violence against women and associated health consequences in Latin America and the Caribbean: a scoping review
Source: Cad Saude Publica. 2026 Oct 30;42:e00048426. doi: 10.1590/0102-311XEN048426 (PMC13722036; doi:10.1590/0102-311XEN048426)
Supplement: Supplementary file 1 [file 1678-4464-csp-42-EN048426-s.pdf]

Supplementary Materials

https://drive.google.com/drive/folders/1o4ce99NQmGEdL-jd1PmWPs3EE-sW_fVj?usp=sharing

Appendix 1 Search Strategy

The search strategy was structured according to the Social Determinants of Health (SDH) framework and aligned with each review question. Searches were conducted in English and Spanish. Controlled vocabulary (e.g., MeSH terms) and free-text terms were adapted to each database (PubMed/MEDLINE, Scopus, and SciELO).

Review Question 1

How have structural and intermediary social determinants of health been conceptualised, operationalised, and empirically analysed in relation to violence against women in Latin America and the Caribbean?

Conceptual Blocks

Block 1. Population

("Women"[MeSH] OR women[tiab] OR woman[tiab] OR adolescent*[tiab])

Block 2. Violence against women

("Violence Against Women"[MeSH] OR "Domestic Violence"[MeSH] OR "Intimate Partner Violence"[MeSH] OR "Sex Offenses"[MeSH] OR "Gender-Based Violence"[tiab] OR "violence against women"[tiab] OR "intimate partner violence"[tiab] OR "dating violence"[tiab] OR "obstetric violence"[tiab])

Block 3. Social determinants

("Social Determinants of Health"[MeSH] OR "Socioeconomic Factors"[MeSH] OR "Social Conditions"[MeSH] OR "Health Inequalities"[MeSH] OR "Psychosocial Factors"[MeSH] OR poverty[tiab] OR education[tiab] OR employment[tiab] OR "gender norms"[tiab] OR machismo[tiab] OR racism[tiab] OR discrimination[tiab] OR migration[tiab])

Block 4. Region

("Latin America"[MeSH] OR "Caribbean Region"[MeSH] OR "South America"[MeSH] OR "Central America"[MeSH] OR "Latin America"[tiab] OR Caribbean[tiab])

Country-level free-text terms included:

(Antigua and Barbuda OR Argentina OR Aruba OR Bahamas OR Barbados OR Belize OR Bolivia OR Brazil OR British Virgin Islands OR Chile OR Colombia OR Costa Rica OR Cuba OR Curaçao OR Dominica OR Dominican Republic OR Ecuador OR El Salvador OR Guatemala OR Guyana

OR Haiti OR Honduras OR Jamaica OR Mexico OR Nicaragua OR Panama OR Paraguay OR Peru OR Puerto Rico OR Saint Kitts and Nevis OR Saint Lucia OR Saint Vincent and the Grenadines OR Suriname OR Trinidad and Tobago OR Uruguay OR Venezuela OR West Indies)

Complete Search String – PubMed

((("Women"[MeSH] OR women[tiab] OR woman[tiab] OR adolescent*[tiab]) AND ("Violence Against Women"[MeSH] OR "Domestic Violence"[MeSH] OR "Intimate Partner Violence"[MeSH] OR "Sex Offenses"[MeSH] OR "Gender-Based Violence"[tiab] OR "violence against women"[tiab] OR "intimate partner violence"[tiab] OR "dating violence"[tiab] OR "obstetric violence"[tiab])) AND ("Social Determinants of Health"[MeSH] OR "Socioeconomic Factors"[MeSH] OR "Social Conditions"[MeSH] OR "Health Inequalities"[MeSH] OR "Psychosocial Factors"[MeSH] OR poverty[tiab] OR education[tiab] OR employment[tiab] OR "gender norms"[tiab] OR machismo[tiab] OR discrimination[tiab])) AND ("Latin America"[MeSH] OR "Caribbean Region"[MeSH] OR "South America"[MeSH] OR "Central America"[MeSH] OR "Latin America"[tiab] OR Caribbean[tiab]))

Complete Search String – Scopus

TITLE-ABS-KEY (women OR woman OR adolescent*) AND TITLE-ABS-KEY ("violence against women" OR "domestic violence" OR "intimate partner violence" OR "gender-based violence" OR "dating violence" OR "obstetric violence") AND TITLE-ABS-KEY ("social determinants" OR "socioeconomic factors" OR "health inequalities" OR poverty OR education OR employment OR "gender norms" OR machismo OR racism OR discrimination OR migration) AND TITLE-ABS-KEY ("Latin America" OR "Caribbean" OR Argentina OR Bolivia OR Brazil OR Chile OR Colombia OR Costa Rica OR Cuba OR Ecuador OR El Salvador OR Guatemala OR Guyana OR Haiti OR Honduras OR Jamaica OR Mexico OR Nicaragua OR Panama OR Paraguay OR Peru OR Puerto Rico OR Suriname OR Uruguay OR Venezuela)

Review Question 2

How do these determinants, individually and in intersection, shape the physical and mental health consequences of violence against women in the region?

The base strategy for Review Question 1 was retained with the addition of a health outcomes block: AND ("Health Status"[MeSH] OR "Mental Health"[MeSH] OR "Depression"[MeSH] OR "Anxiety Disorders"[MeSH] OR "Cardiovascular Diseases"[MeSH] OR "Maternal Health"[MeSH] OR "Self-Rated Health"[tiab] OR depression[tiab] OR anxiety[tiab] OR PTSD[tiab] OR suicid*[tiab])

SciELO Searches

In SciELO, searches were conducted primarily in Spanish, given the predominance of Spanish-language journals indexed in Latin America. Equivalent Spanish terms were used for violence (e.g., 'violencia contra la mujer', 'violencia de pareja', 'violencia obstétrica'), social determinants

('determinantes sociales', 'desigualdad social', 'pobreza', 'educación', 'empleo'), and health outcomes ('salud mental', 'depresión', 'ansiedad', 'salud materna'). Boolean operators and syntax were adapted to the SciELO interface.
